# ULK1 Inhibition as a Targeted Therapeutic Strategy for Psoriasis by Regulating Keratinocytes and Their Crosstalk With Neutrophils

**DOI:** 10.3389/fimmu.2021.714274

**Published:** 2021-08-04

**Authors:** Xiaonan Qiu, Lin Zheng, Xiuting Liu, Dan Hong, Mintong He, Zengqi Tang, Cuicui Tian, Guozhen Tan, Sam Hwang, Zhenrui Shi, Liangchun Wang

**Affiliations:** ^1^Department of Dermatology, Sun Yat-sen Memorial Hospital, Sun Yat-sen University, Guangzhou, China; ^2^Institute of Dermatology, Chinese Academy of Medical Science and Peking Union Medical College, Nanjing, China; ^3^Department of Dermatology, University of California, Davis, Sacramento, CA, United States

**Keywords:** ULK1 (unc-51 like autophagy activating kinase 1), psoriasis, keratinocyte, neutrophil, autophagy

## Abstract

Psoriasis is a common inflammatory skin disease resulting from an interplay of keratinocytes and immune cells. Previous studies have identified an essential role of autophagy in the maintenance of epidermal homeostasis including proliferation and differentiation. However, much less is known about the role of autophagy-related proteins in the cutaneous immune response. Herein, we showed that ULK1, the key autophagic initiator, and its phosphorylation at Ser556 were distinctively decreased in the epidermis from lesional skin of psoriasis patients. Topical application of SBI0206965, a selective ULK1 inhibitor, significantly attenuated epidermal hyperplasia, infiltration of neutrophils, and transcripts of the psoriasis-related markers in imiquimod (IMQ)-induced psoriasiform dermatitis (PsD). *In vitro*, ULK1 impairment by siRNA and SBI0206965 arrested cell proliferation and promoted apoptosis of keratinocytes but had a marginal effect on the expression of proinflammatory mediators under steady status. Surprisingly, SBI0206965 blocked the production of chemokines and cytokines in keratinocytes stimulated by neutrophils. Of interest, the pro-apoptotic and anti-inflammatory effects of ULK1 inhibition cannot be fully replicated by autophagic inhibitors. Our findings suggest a self-regulatory process by downregulating ULK1 to maintain the immune homeostasis of psoriatic skin *via* regulating keratinocytes and their crosstalk with neutrophils, possibly through both autophagy-dependent and independent mechanisms. ULK1 might be a potential target for preventing or treating psoriasis.

## Introduction

Psoriasis is characterized by keratinocyte hyperproliferation and infiltration of immune cells in inflamed skin (1). Keratinocytes (KCs) not only are the target cells but also plays a pivotal role in psoriasis development (2). At the initiating phase, keratinocytes overproduce antimicrobial peptides (AMP), cytokines of the IL-1 family together with chemokines to recruit and activate innate immune cells such as neutrophils, mast cells, macrophages, and plasmacytoid dendritic cells (pDC) in response to the upstream triggers (3, 4). Infiltrated immune cells, in return, stimulate KCs, which undergo dysregulated proliferation and differentiation, and at the same time, produce more chemokines (such as CCL20, CXCL1, CXCL2 and CXCL8) and AMP (such as S100 proteins and β-defensins) to magnify the immune circuits responsible for the induction and maintenance of psoriasis. Among the infiltrated immune cells, neutrophils accumulate into the epidermis, forming Munro microabscesses, which serve as a typical histopathologic hallmark of psoriasis (5). Neutrophils could stimulate keratinocytes by Toll-like receptors (TLRs), oxidative stress, granular components, and neutrophil extracellular traps (NETs) (6, 7). NETs further supply IL-17 and induce T helper 17 (Th17) cells to release more cytokines, therefore bridging the innate immune and adaptive immune systems (8). Indeed, neutrophil-keratinocyte crosstalk is an early target of IL-17A antibody-mediated therapies in psoriasis (9), suggesting that communication of keratinocytes and neutrophils is involved in the immunopathogenesis of psoriasis.

Macroautophagy (hereafter referred to as autophagy), the intracellular self-digestion process, plays a pivotal role in maintaining energy homeostasis and protein synthesis both in physiological and pathological conditions (10, 11). Increasing evidence shows that autophagy regulates cell proliferation, differentiation, and antimicrobial defense of KCs (12, 13), and therefore could be involved in some skin diseases with epidermal hyperplasia such as psoriasis. Indeed, autophagy-related proteins such as LC3, Beclin 1, and ATG5 have been reported to be dysregulated in psoriatic epidermis (14, 15). A recent study showed that keratinocyte-specific ablation of autophagy caused resistance to imiquimod (IMQ)-induced psoriasiform dermatitis (PsD), further suggesting a pathogenic role of autophagy in psoriatic KCs (16). The Unc-51 like autophagy activating kinase 1 (ULK1) complex plays a central role in the initiation stage of autophagy (17). Upon activation, ULK1 induces the next step of autophagy, nucleation of the immature autophagosome, by phosphorylating the downstream BECN1 complex. Dysregulation of ULK1 has been implicated in numerous diseases such as cancer (18–20), diabetic disease (21), and neurodegeneration (22), mostly *via* regulation of autophagy. A few studies proposed a link between ULK1 gene polymorphisms and inflammatory diseases like Crohn’s disease (23) and ankylosing spondylitis (24). Still, how ULK1 mechanistically regulates inflammation remains to be elucidated. Even much less is known about the role of ULK1 in inflammatory skin disease like psoriasis.

Herein, we reported that both ULK1 and its functional form of phosphorylated ULK1 (pULK1) was downregulated in the lesional epidermis from psoriasis patients. Abrogation of ULK1 kinase activity utilizing the ULK1-specific kinase inhibitors SBI0206965 ameliorated PsD induced by IMQ in both preventative and therapeutic manner. The downregulation of ULK1 and pULK1 shrunk the keratinocytes population by decreasing proliferation and increasing apoptosis as well as blocked proinflammatory mediators produced by KCs in response to neutrophils stimulation. Unexpectedly, the pro-apoptotic and anti-inflammatory effects were unlikely to be autophagic-related since these functions cannot be replicated by autophagy inhibitors. Our findings highlight the role of ULK1 in regulating intrinsic functions of KCs but more interestingly in the KCs-neutrophil crosstalk *via* both autophagy-dependent and independent manner. Inhibitors of ULK1 may provide a novel therapeutic option for the treatment of psoriasis.

## Materials and Methods

### Human Skin Specimen

For skin specimens, lesional skin samples were collected from patients with psoriasis vulgaris or eczema. The diagnosis was based on clinical history, skin manifestation, and histologic examination. Normal skin samples were gathered from the peripheries of removed nevi. Non-lesional skin from psoriasis patients were taken at least five centimeters from a plaque. The study was approved by the research ethics board of Sun Yat-sen Memorial Hospital and informed consent of donors was obtained.

### Microarray Experiments

Gene-expression profiles were analyzed with the Affymetrix GeneChip Human Genome U133 Plus 2.0. The target preparation, library labeling, hybridization, post-wash, and signal scanning were performed by CapitalBio Biotechnology (Beijing, China).

### Immunohistochemistry

Formaldehyde-fixed, paraffin-embedded skin samples were stained with H&E using standard procedures. Epidermal thickness was measured with a computer-assisted quantitative image analysis software (ImageJ).

Immunohistochemistry was performed as we previously described. Briefly, paraffin-embedded tissue sections (4 mm) from human and mice were deparaffinized with xylene, rehydrated, and subjected to heat-induced epitope retrieval methods before incubation with the appropriate antibody. The next day, sections were incubated with appropriate horseradish peroxidase (HRP) conjugated secondary antibody, developed in 3,3’-diaminobenzidine (DAB) kit, and counterstained with hematoxylin.

The quantification was performed using Image pro plus 6.0 software (Meyer Instruments, Inc.). IOD (integrated optical density) is defined as the sum of the quantity of the positive material of all the positions in the region. The average intensity/density in the selected area was calculated as IOD/Area.

### IMQ-Induced Psoriasis Model

Gender and age-matched C57BL/6 mice between 8 and 12 weeks of age were purchased from the Sun Yat-sen University laboratory animal center (Guangzhou, China) or The Jackson Laboratory (Bar Harbor, ME). To induce psoriasis-like lesions, mice received a daily topical dose of 62.5 mg of imiquimod cream (5%) (3M Pharmaceutical, Maplewood, Minnesota, U.S) on the shaved back consecutively for 6 days, as described previously (25). 200µl SBI-0206965 (MedChemexpress, Princeton, NJ, USA) at 100 µM and 200 µM (dissolved in acetone/corn oil 1:4 v/v) was topically applied to dorsal skin 2 days before imiquimod application for 8 consecutive days. For the preventative and therapeutic model, SBI-0206965 at concentration 200 µM were applied as indicated period. Mice were treated with SBI in the morning (8AM) and 8 hours later, treated with IMQ (4PM). All experiments were performed according to the animal care and use committee guidelines of Sun Yat-sen University.

The psoriasis severity index (PSI) scoring system was utilized to rate the macroscopic appearance. Erythema, scaling, and thickening was scored independently on a scale from 0 to 4: 0, none; 1, slight; 2, moderate; 3, marked; 4, very marked. The cumulative score of erythema, scaling, and thickening on a scale of 0 to 4 was summed up daily for each animal.

### Keratinocyte Culture

HaCat cells were supplied by the Institutes of Biomedical Sciences (IBS) (Fudan University, China) and maintained in an incubator at 37°C in a humidified atmosphere containing 5% CO_2_ plus 95% air. The cells were grown in DMEM medium containing 10% fetal bovine serum. Medium was refreshed every 2 days, and cells were sub-cultured according to the cell fusion.

Primary keratinocytes (PCS-200-010, ATCC) were cultured in Dermal Cell Basall Medium (PCS-200-030, ATCC) supplemented with Keratinocyte Growth Kit (PCS-200-040, ATCC), 10U/mL of penicillin and 10µg/mL of streptomycin and 25ng/mL of amphotericin (03-033-1B, BI). All the cells were cultured in a 37°C, 5% CO_2_, humidified incubator.

### Keratinocytes Transfected With siRNAs

To knock down ULK1, ULK1-siRNA or non-coding siRNA (NC-siRNA) (GenePharma, Shanghaia) was transfected with Lipofectamine RNAiMax (Thermo Fisher Scientific, Waltham) into keratinocytes at a concentration of 60nM.

### Coculture System of Keratinocyte and Neutrophils

7 ml of human whole blood was treated with EDTA then added to 6 ml of Polymorphprep™(Proteogenix) in a 15 ml centrifuge tube. The samples were centrifuged at 400xg for 30 minutes at room temperature. After centrifugation, The neutrophils then existed as the lower bands and were harvested using Pasteur pipettes.

Neutrophils were resuspended with DMEM medium containing 10% fetal bovine serum with 10 uM SBI-0206965 (MedChemexpress, Princeton, NJ, USA) or DMSO of the same concentration. The neutrophil suspension was then added to HaCat cells and co-cultured for 24 hours in a humidified incubator at 37°C containing 5% CO2. The ratio of neutrophil to HaCat was 3.75 to 1.

### RNA Extraction

Total RNA was extracted using EZ-press RNA Purification Kit for cultured keratinocytes (EZBioscience, B0004DP) or RNeasy Fibrous Tissue Mini Kit for animal tissue (QIAGEN, 74704). For cultured cells, cells seeded in 12-well plates were lysed using lysis buffer in the kit followed by ½ volume of ethanal. The mixture was added into columns and processed to centrifugation. The RNA bound to column membrane was eluted then. For each well, about 10 ug RNA was yielded. For animal tissue, the tissue was cut up, then Buffer RLT and proteinase K were added. After centrifugation, the supernatant was mixed with ethanal and centrifuged through columns. DNA left was removed by DNase treatment. RNA bound to the column membrane was eluted. For 30 mg tissue, about 20 ug RNA could be yielded.

### Quantitative Real-Time PCR

Total RNA was extracted from keratinocytes using RNAiso Plus reagent (Takara, Shiga, Japan). Total RNA of mouse ear skin was extracted by using an RNeasy Fibrous Tissue Kit (Qiagen, Hilden, Germany). Quantitative real-time PCR was performed using a ViiA 7 Real-Time PCR system or Quant Studio 3 real-time PCR system (Applied Biosystems, Foster City, CA, USA). For the analysis of animal studies, the data were present as 2-ΔΔCT (2^(ΔCT(a target sample)−ΔCT(a reference sample)). For the analysis of *in vitro* studies, the data were present as 2-ΔCt (2^housekeeping gene/2^gene of interest). The primers were obtained from Integrated DNA Technologies, Inc (Skokie, IL, USA) or synthesized according to the sequence from the Primerbank database. Detailed Catalogue number or sequence of prier is available in **Supplementary Documents**.

### Protein Extraction and Western Blot

Cultured cells were washed twice with ice cold PBS then RIPA buffer (CWBIO, CW2333S) with protease and phosphatase inhibitors added (Beyotime, P1045). The cells were scratched into Eppendorf tubes and vortexed. After centrifugation, the supernatant was collected. The protein concentration was evaluated using Pierce BCA Protein Assay Kit (ThermoFisher, 23227). Then SDS (Beyotime, ST628) and RIPA buffer were added into the solution followed by boiling for 10 minutes. The immunoblot analysis was performed according to the manufacturer’s instruction (Beyotime, P0012A). After detection of phosphor-ULK1, the membrane was stipped (Abcam, ab270550) for 20 minutes on rotator before blotting for ULK1. Primary antibodies and concentrations: anti-phosphor-ULK1 (Abcam, ab203207, 1:1000); anti-ULK1 (Abcam, ab128859, 1:1000); anti-GAPDH (Cell Signaling Technology, 5174, 1:1000); anti-p62 (Cell Signaling Technology, 5114, 1:1000); anti-LC3 (Cell Signaling Technology, 2775S, 1:1000). The intensities of the bands were quantified using ImageJ software.

### Statistical Analysis

All data are shown as mean ± SEM. Data were analyzed using GraphPad Prism version 6 (GraphPad Software, San Diego, CA, USA). A two-sided unpaired Student’s t-test was used to compare two groups, and a one-way analysis of variance (ANOVA) Bonferroni *post hoc* test was used for multiple comparisons. Two-way ANOVA with Tukey *post hoc* test was used to analyze the curves of PSI score. A *P*-value less than 0.05 was considered statistically significant.

More detailed experimental procedures are described in the **Supplementary Documents**.

## Results

### Characterization of ULK1 Expression in Skin Lesions From Patients With Psoriasis

**Figure 1:**
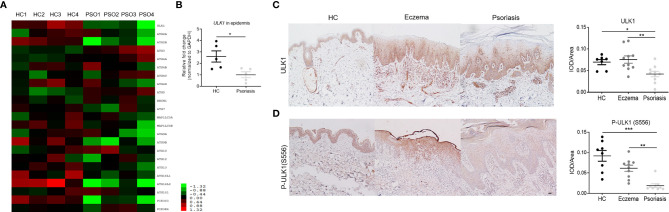
Decreased expression of ULK1 in psoriatic epidermis. **(A)** Heatmap of the autophagy-related gene expression determined by microarray (n=4). **(B)** Expression of ULK1 mRNA (n=5) in epidermis from psoriatic lesions and normal skin from health controls (HC). **(C, D)** Representative image and quantification of ULK1 and phospho-ULK1 (Ser556) expression by immunohistochemical (IHC) staining in healthy control, lesional skin from patients with eczema or psoriasis (n=8-10). Scale bars, 50 μm. Data are presented as mean± SEM. *p < 0.05; **p < 0.01; ***p < 0.001.

We first applied mRNA microarray to determine the expression of autophagy-related molecules in the epidermis from healthy controls and psoriatic lesions. Among the majority of autophagy-related genes, ULK1 (fold change=0.42, q-value=0.0051), ATG2B (fold change=0.37, q-value=0.0051), ATG9B (fold change=0.46, q-value=0.0063), ATG16L2 (fold change=0.27, q-value=0.0025), and PIK3C3 (fold change=0.50, q-value=0.02) were significantly downregulated, whereas ATG5 (fold change=1.52, q-value=0.018) was upregulated in the psoriatic epidermis compared to healthy controls (**Figure 1A**). The expression of the other autophagy-related genes was not different in the skin between psoriasis patients and healthy controls. The decrease of ULK1 mRNA expression in the psoriatic epidermis was further confirmed by quantitative PCR (qPCR) (**Figure 1B**). As for the pattern of distribution, ULK1 was thoroughly expressed in all epidermal layers in the skin from healthy controls and eczematous lesions as determined by immunohistochemical (IHC) staining. However, it was mainly observed in the dermal side, whereas greatly reduced in the epidermal side in the epidermis of psoriatic lesions (**Figure 1C**) [F (2, 24) = 6.599, p=0.0052]. Notably, ULK1 was abundantly expressed on the cellular infiltrate in the psoriatic and eczematous dermis but scarcely scattered in the dermis from healthy donors. ULK1 was specifically involved in autophagy by integrating the upstream signals of AMP-activated protein kinase (AMPK) and transducing them to the downstream autophagy pathway (26). The phosphorylation of ULK1 at Ser556 site (Ser556 in human and Ser555 in murine cells) was AMPK-dependent, which is required for the activation of downstream autophagy pathway (27). In lines with total ULK1 expression, the phosphorylation of ULK1 at Ser556 was even more profoundly reduced in psoriatic lesions than in healthy controls and eczematous lesions (**Figure 1D**) [F (2, 24) = 16.93, p<0.0001]. Albeit not statistically significant, IHC analysis further suggested a trend towards lower levels of total ULK1 and phosphorylation of ULK1 in the non-lesional epidermis from psoriasis patients *versus* normal epidermis from healthy donors (**Figure S1**). Collectively, both ULK1 and the activation form of phosphorylated-ULK1 were dysregulated in psoriatic skin compared to healthy skin.

### Inactivation of the Kinase Activity of ULK1 by SBI0206965 Ameliorated PsD Induced by IMQ

**Figure 2:**
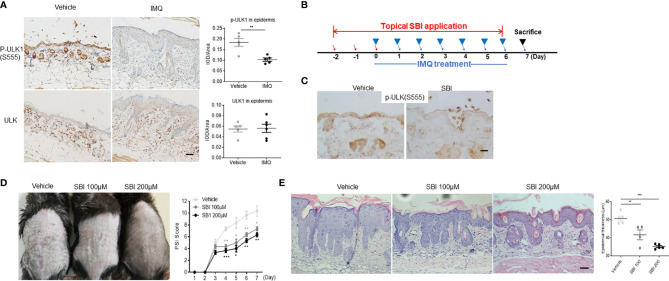
ULK1 inhibitor SBI-0206965 (SBI) ameliorates psoriasiform dermatitis (PsD) induced by imiquimod (IMQ). **(A)** Representative images and quantification of immunochemical staining of p-ULK1 (ser555) and ULK1 in dorsal skin from mice treated with topical IMQ or vanicream for 7 days (scale bars, 50 μm). **(B)** Schematic illustration of experimental protocols. **(C)** p-ULK1 (ser555) expression in dorsal skin from mice treated with 100 µM SBI as shown in **(B)** on day 0 (scale bars, 25 μm). **(D)** Manifestations and the severity of skin inflammation measured by PSI score in mice treated as shown in **(B)**. **(E)** HE staining and histological analysis of epidermis thickness (scale bars, 50 μm). Data are presented as mean± SEM. *p < 0.05; **p < 0.01; ***p < 0.001.

We then adopted the IMQ-induced psoriasis model to investigate whether dysregulation of ULK1 was involved in the pathogenesis of psoriasis. First of all, we examined ULK1 expression in psoriatic lesions induced by IMQ and found that the phosphorylation of ULK1 at Ser555 site was remarkably reduced in the epidermis whereas the total protein of ULK was comparable to mice received vanicream, which was partially similar to what was observed in human (**Figure 2A**). Therefore, we treated the mice with topical application of SBI0206965 (SBI), a selective ULK1 kinase inhibitor (28), 2 days before the initial IMQ treatment (**Figure 2B**) and ensured that the phosphorylation of ULK1 at Ser555 was repressed in the skin as compared to mice receiving vehicle (**Figure 2C**). We then continuously treated mice with SBI together with IMQ and measured skin inflammation by psoriasis severity index (PSI). Those mice developed less severe skin inflammation beginning on day 4 compared to vehicle-treated mice (**Figure 2D**) [F (2, 6) = 34.33, p=0.0005]. At the end of the experiment on day 7, mice received SBI treatment displayed less scaly, erythematous skin lesions. Consistently, histologic analysis revealed attenuated epidermal hyperplasia and hyperkeratosis in a dose-dependent manner of SBI (**Figure 2E**) [F (2, 11) = 25.03, <0.0001]. Together, these findings suggested that the downregulation of phosphorylated ULK1 limited psoriasis-like skin inflammation induced by IMQ.

### Topical Application of SBI Improves PsD in Both Therapeutic and Preventive Manners With Inhibited Infiltration of Neutrophils

**Figure 3:**
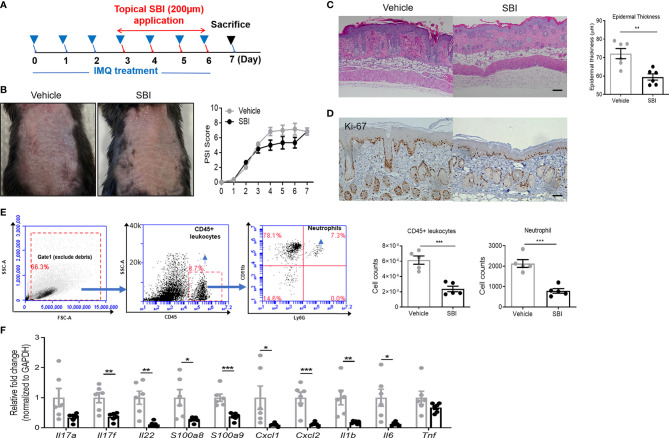
Topical application of SBI therapeutically improves PsD with inhibited infiltration of neutrophils. **(A)** Schematic illustration of experimental protocols. **(B)** Manifestations and the severity of skin inflammation measured by PSI score in mice treated as shown in **(A)**. **(C)** HE staining and histological analysis of epidermis thickness. **(D)** Ki-67 staining examined by immunohistochemistry staining. **(E)** Flow cytometry plots showing gating strategy. Absolute number of infiltrating CD45+ cells and neutrophils examined by flow cytometry. **(F)** mRNA expression of cytokines in the whole ear. 6 animals per group. Scale bars, 50 μm. Data are presented as mean ± SEM. *p < 0.05; **p < 0.01; ***p < 0.001.

To assess whether or not SBI treatment has therapeutic effects on psoriasis-like inflammation, mice first received IMQ for three days and underwent SBI treatment together with IMQ for the last four days (**Figure 3A**). On day 3 after IMQ application, the skin inflammation was already established as evidenced by the PSI score. Albeit not statistically significant, a trend towards a lower PSI score was observed in the SBI-treated group from day 4 to day 6 (**Figure 3B**). Strikingly, histological analysis showed that SBI treatment ameliorated the epidermal hyperplasia as measured by the decreased epidermal thickness (**Figure 3C**) and the dampened nuclear staining of proliferation marker Ki-67 (**Figure 3D**). Flow cytometric analysis showed a reduction in the infiltration of CD45+ leukocytes and neutrophils (**Figure 3E**). Whilst the infiltration of CD3+ T cells were comparable between mice treated with vehicle or SBI (**Figure S1A**). Molecular analysis demonstrated a significant reduction in the transcripts of psoriasis-related proinflammatory mediators, including Th17 cytokines (*Il17f, Il22*), neutrophil chemoattractants (*Cxcl1, Cxcl2*), AMPs (*S100a8, S100a9*) and proinflammatory cytokines (*Il1b, Tnf*) (**Figure 3F**).

We then examined whether or not a short-term pre-treatment with SBI was sufficient to prevent psoriasis-like inflammation. To do that, mice were only consecutively treated with SBI for four days that was initiated two days ahead of IMQ application (**Figure S3A**). Mice pretreated with SBI developed less severe psoriatic inflammation that was evidenced by less scaly erythema and significantly lower PSI score (**Figure S3B**). Similar to the therapeutic model, administration of SBI significantly blocked epidermal thickening (**Figures S3C, D**) and inhibited infiltration of neutrophils but not CD3+ T cells (**Figures S3E, F**). Consistent with these findings, most psoriasis-related cytokines were also greatly reduced in mice pretreated with SBI (**Figure S3G**), especially *Cxcl1, Cxcl2, S100a8* and *Il6*. Thus, topical application of ULK1 inhibitor SBI improved IMQ-mediated dermatitis in both a preventative and therapeutic manner with less infiltration of neutrophils.

### Inhibition of ULK1 Impaired the Function of Keratinocytes *In Vitro*

**Figure 4:**
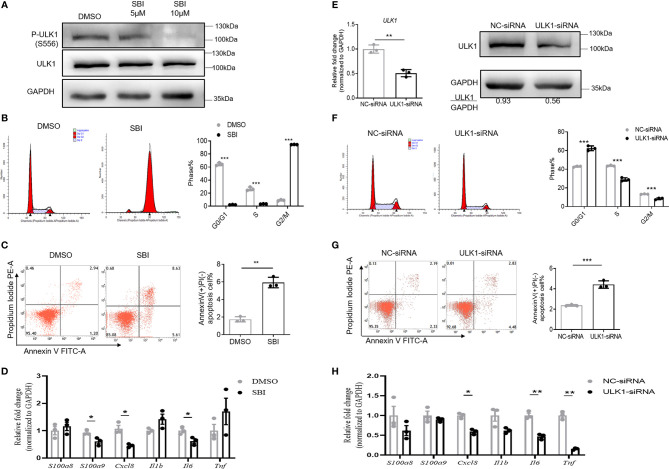
Inhibition of ULK1 suppresses proliferation and promotes apoptosis of keratinocytes *in vitro*. **(A)** Immunoblot of HaCat keratinocytes 24 hours after cocultured with DMSO, 5 µM or 10 µM SBI-0206965 (SBI). **(B)** Cell cycle analysis of HaCat keratinocytes treated with DMSO or 10 µM SBI for 24 hours. The bar graph shows the percentage of cell population in each phase of cell cycle. **(C)** Apoptosis of HaCat keratinocytes 24 hours after serum deprivation in the presence of DMSO or 10 µM SBI. **(D)** mRNA expression of psoriasis-related inflammatory mediators in HaCat keratinocytes cocultured with DMSO or 10 µM SBI for 24 hours. **(E)** mRNA and protein expression of ULK1 in HaCat keratinocytes transfected with negative control-siRNA(NC-siRNA) and ULK1-siRNA. **(F)** Cell cycle analysis of HaCat keratinocytes 72 hours after transfection with NC-siRNA or ULK1-siRNA. **(G)** Apoptosis of transfected HaCat keratinocytes 24 hours after serum deprivation. **(H)** mRNA expression of psoriasis-related inflammatory mediators in HaCat keratinocytes transfected with NC-siRNA or ULK1-siRNA for 72 hours. Data are presented as mean ± SEM. *p < 0.05; **p < 0.01; ***p < 0.001.

We then characterized the phenotype and function of keratinocytes with impaired ULK1 activation. To this end, we treated HaCat cells, a human keratinocyte cell line, with SBI. SBI did not change ULK1 expression (112 kDa) but inhibited the kinase activity of ULK1 by decreasing the phosphorylation of ULK1 at Ser556 (112 kDa) in keratinocytes relative to GAPDH (37 kDa) (**Figure 4A**). The inhibition of the kinase activity of ULK1 by SBI delayed the mitosis of keratinocytes that was evidenced by the accumulation of keratinocytes in the G2/M phase (**Figure 4B**). Besides, the apoptotic keratinocytes were dramatically increased in SBI transfected keratinocytes in response to serum depletion (**Figure 4C**). In contrast to what we observed *in vitro* studies, however, treatment of SBI only led to a marginal decrease in the transcripts of *CXCL8*, *S100A9*, and *IL6* in keratinocytes and did not influence *S100A8*, *IL1B*, and *TNF* (**Figure 4D**).

To investigate whether the reduction of ULK1 expression in keratinocytes participates in psoriasis development, we further transfected HaCat cells with small interfering RNA against ULK1 (ULK1-siRNA) or non-targeting negative control siRNA (NC-siRNA). As expected, the expression to ULK1 was efficiently downregulated both at mRNA and protein levels in keratinocytes transfected with ULK1-siRNA as compared to those with NC-siRNA (**Figure 4E**). With the impaired expression of ULK1, the proliferation of keratinocytes was greatly reduced, which was evidenced by the decrease of the proportion of keratinocytes at S and G2/M phases (**Figure 4F**). Similar to what was observed in keratinocytes treated with SBI, knockdown of ULK1 led to an increased apoptotic rate (**Figure 4G**) and slight reductions in mRNA expression of *IL6* and *TNF* but did not affect other proinflammatory markers (**Figure 4H**). Taken together, the results suggest that inhibition of ULK1 impairs proliferation and induces apoptosis of keratinocytes.

We then examined whether the above findings could be reproduced by using primary human keratinocytes (PHK), a research model with more cutaneous biological features than HaCat cell line. Suppression of ULK1 enzymatic activity with SBI inhibitor led to restriction of PHK proliferation and the upregulation of apoptotic rate, which were similar to what was observed in HaCat cell line (**Figures S4A–C**). SBI treatment has little effect on the expression of proinflammatory markers in PHK under steady status and even slightly promoted the inflammation under stimulation of IL-17A (**Figure S4D**). Inhibition of ULK1 gene expression with ULK1-siRNA resulted in a lower population of PHK at S-phase, indicating a restriction of cell proliferation. Whereas the apoptosis of PHK was not affected, which was different from what was observed in HaCat cell line (**Figures S4E–G**). Downregulation of ULK1 with siRNA did not affect the expression of most psoriasis-related cytokines in PHK without stimulation but increased the transcripts of TNF and CXCL8 in the presence of IL-17A (**Figure S4H**). Overall, these findings demonstrated that the impacts of ULK1 dysfunction on HaCat cell line in terms of proliferation and apoptosis were mostly reproduced on PHK.

### Inactivation of ULK1 Impaired the Crosstalk Between Keratinocytes and Neutrophils

**Figure 5:**
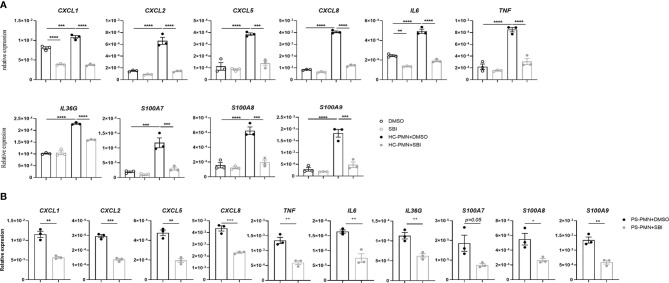
Inactivation of ULK1 by SBI suppressed the inflammation in keratinocytes stimulated by neutrophil. **(A)** mRNA expression of psoriasis-related inflammatory mediators by keratinocytes stimulated with neutrophils isolated from healthy donors (HC) or **(B)** psoriasis patients in the presence of 10 µM DMSO or SBI for 10 hours. Data are representative of three independent experiments. Data are presented as mean ± SEM. *p < 0.05; **p < 0.01; ***p < 0.001; ****p < 0.0001.

The aberrant crosstalk between immune cells like neutrophils and resident keratinocytes generates inflammatory circuits responsible for the initiation, progression, and persistence of the disease (7). Our data showed that SBI administration failed to decrease the expression of proinflammatory mediators in keratinocytes *in vitro* but profoundly suppressed the majority of psoriasis-related cytokines in animal studies. Given the above observations, we hypothesize that SBI may block the inflammation by inhibiting the interaction between keratinocytes and neutrophils. To test this theory, we first cocultured keratinocytes with neutrophils isolated from healthy donors in the presence or the absence of SBI. Interaction with neutrophils induced expression of a variety of inflammatory mediators in keratinocytes previously reported to be upregulated in psoriatic epidermis (29), such as CXCL1, CXCL2, CXCL5, CXCL8, IL‐6, TNF‐α, IL‐36G, and S100A7-9 (**Figure 5A**) [CXCL1, F (3, 8) = 157.4; CXCL2, F (3, 8) = 76.40; CXCL5, F (3, 8) = 46.10; CXCL8, F (3, 8) = 1000; IL‐6, F (3, 8) = 157.9; TNF‐α, F (3, 8) = 62.68; IL‐36G, F (3, 8) = 181.3; S100A7, F (3, 8) = 31.53; S100A8, F (3, 8) = 43.05; S100A9, F (3, 8) = 48.55]. Strikingly, All the elevated expression of inflammation markers in keratinocytes were suppressed by administration of SBI. No obvious difference in the production of proinflammatory mediators was found when we stimulate KCs with neutrophils from healthy donors or psoriasis patients (data not shown). Still, SBI exerted a similar suppressive effect on KCs stimulated with neutrophils from psoriasis patients (**Figure 5B**). We then asked if SBI targets neutrophils and indirectly blocks the inflammation of keratinocytes. To this end, we first tested the expression levels of ULK1 on neutrophils between healthy donors and psoriasis patients. Contrary to what we observed in the epidermis, the mRNA levels of ULK1 on neutrophil was comparable between healthy donors and psoriasis (**Figure S5A**). We were also unable to detect phosphorylation of ULK1 on neutrophils from either healthy subjects or psoriasis patients using western blot (data not shown). Furthermore, pretreatment of neutrophils with SBI before co-culturing failed to block the inflammatory response in KCs (**Figure S5B**). However, we observed a positive correlation of ULK1 with myeloperoxidase (MPO), a major protein constituent of granules, in neutrophils from both healthy donors and patients with psoriasis. An additional correlation of ULK1 with other granule-derived antimicrobial peptides and enzymes, including Cathelicidin (LL37) and elastase (ELANE), was found exclusively in psoriasis patients but not health donors (**Figure S6**). Together, these data suggest that inactivation of ULK1 is likely to inhibit inflammation by targeting KCs and impairing their crosstalk with neutrophils.

### Autophagy Inhibitor Failed to Replicate the Effect of ULK1 Inhibition

**Figure 6:**
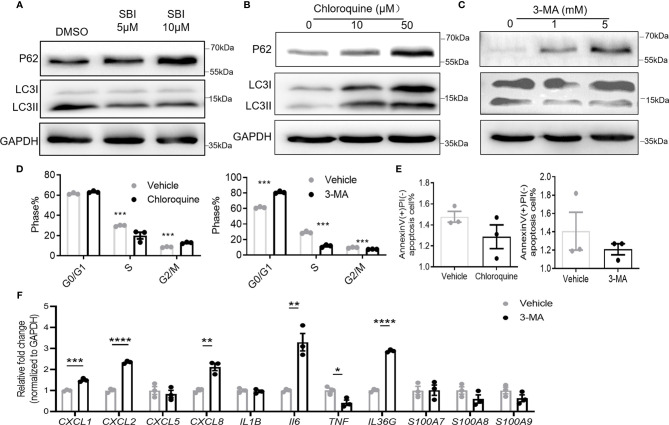
Autophagy inhibitors fail to fully replicate the effect of ULK1 inhibition on keratinocyte. **(A–C)** Expression of p62 and LC3 I/II in HaCat keratinocytes 24 hours after incubation with SBI0206965 (SBI) **(A)** or chloroquine **(B)** or 3-methyladenine(3-MA) at indicated concentration **(C)**. **(D)** Cell cycle analysis and **(E)** apoptosis of keratinocytes after 24 hours with the treatment of 10 µM chloroquine or 5 mM 3-MA. **(F)** mRNA expression of psoriasis-related inflammatory mediators by keratinocytes cocultured with neutrophils from healthy donors in the presence of 5 mM 3-MA. Data are representative of three independent experiments. Data are presented as mean ± SEM. *p < 0.05; **p < 0.01; ***p < 0.001; ****p < 0.0001.

Given the pivotal role of ULK1 in autophagy, we asked if these anti-psoriatic effects were autophagy-dependent. Treatment with SBI slightly compromised the autophagy of keratinocytes with the accumulation of autophagy substrate of p62 (62kDa) and reduction of the conversion of LC3B I (14kDa) to LC3B II (16kDa) (**Figure 6A**). Chloroquine and 3-methyladenine (3-MA) are two commonly used autophagy inhibitors that inhibit autophagy by blocking the fusion of autophagosome and lysosome and inhibiting the phosphatidylinositol 3-kinases (PI3K) to forbid the formation of autophagosome, respectively (30). To investigate whether ULK1 mediated the function of keratinocytes through autophagy, we treated keratinocytes with the two autophagic inhibitors. Treatment with chloroquine resulted in accumulation of p62 and LC3, whereas 3-MA led to an increase of p62 and decrease of LC3 II/LC3 I (**Figures 6B, C**). Both chloroquine and 3-MA treatment efficiently inhibited the proliferation of keratinocytes as evidenced by the lower percentage of cells in S phase (**Figure 6D**). However, unlike what was observed in keratinocytes by inhibiting ULK1, chloroquine and 3-MA treatment failed to induce apoptosis in response to serum depletion (**Figure 6E**). Nevertheless, the treatment of 3-MA increased the expression of *CXC1*, *CXCL2*, *CXCL8*, *IL6* and *IL36G* in contrary to the anti-inflammatory effect induced by SBI in keratinocytes stimulated with neutrophils (**Figure 6F**). Results from PHK confirmed the above findings (**Figure S7**). We further examined the effects of 3-MA on PHK in the presence of IL-17A. Treatment of 3-MA greatly increased the transcripts of some proinflammatory mediators including *CXCL2*, *IL36G*, *S100A8* and *S100a9* but moderately suppressed the expression of *CXCL1* and *IL1B* (**Figure S8**). Together, these results indicated that ULK1 regulated keratinocytes’ function not only through autophagy but also through an autophagy-independent pathway.

## Discussion

Emerging evidence showed that autophagy is essential for skin differentiation, development, and survival (31). However, studies on ULK1, the essential autophagic initiator are limited. Our data showed that ULK1 inhibition suppressed proliferation and induced apoptosis of keratinocytes. These findings were consistent with what was reported in many cancer cell lines where ULK1 inhibitors exerted oncogenic activity (32, 33). In our present study, increased apoptosis of keratinocytes was observed by knockdown of ULK1 and treatment with ULK1 inhibitor but not by directly inhibiting autophagy with chloroquine or 3-MA, suggesting that ULK1 mediated apoptosis in an autophagy-independent manner. Indeed, previous literature indicates ULK1 inhibitors induced cell apoptosis *via* caspase activation and dysregulation of Bcl2/Bcl-xl (33–35). In agreement, we also observed a decrease in transcripts of Bcl2 and Bcl-xl in KCs treated with SBI (data not shown).

Of greater interest is our findings that SBI strongly suppressed the inflammatory response in keratinocytes cocultured with neutrophils. Neutrophils are recruited to psoriasis lesions, particularly in the epidermis where they cluster to form spongiform pustules of Kogoj in the stratum spinosum and Munro’s microabscesses in the stratum corneum (6). Infiltrated neutrophils undergo NETosis (The process of NET formation) in close proximity to the epidermis and stimulate KCs to produce high levels of various inflammatory mediators, including CXCL1,CXCL2, CXCL8 and IL-36γ, which sustains the formation of NETosis and promotes the recruitment of neutrophils (7, 25, 36). Our data showed that SBI failed to affect expression of proinflammatory markers in keratinocytes under steady state or exposure to IL-17A but significantly blocked the inflammation when stimulated by neutrophil, suggesting that ULK1 inhibitor may specifically targets the communication between keratinocytes and neutrophils. Our data revealed a positive correlation between ULK1 and NETs components including MPO, LL37 and elastase (37), suggesting a potential role of ULK1 in regulating NETosis. Indeed, several lines of evidence both in human system and in murine models propose a critical role for autophagy in neutrophil functions, including reactive oxygen species production and release of NETs (7, 38). Surprisingly, the anti-inflammatory effect by ULK1 inhibitor cannot be replicated by classic autophagy inhibitor, 3-MA. These findings indicated an autophagy-independent role of ULK1 in regulating immunological signaling. Indeed, a central role of ULK1 in interferon (IFN)-dependent immunity was described recently (39). Upon IFN stimulation, phosphorylation of ULK1 was required for phosphorylation of p38 MAPK and transcription of IFN-stimulated genes in multiple cells. Further studies are required to elucidate the mechanisms of ULK1 regulating cytokine production under the settings of psoriasis.

Our results showed that ULK1 inhibition exerted an anti-psoriatic effect. However, psoriatic KCs already exhibited a lower level of ULK1 as well as phosphorylation at Ser556, implicating that a self-regulatory process exists to downregulate ULK1 in maintaining epidermal homeostasis in the context of psoriasis. This hypothesis was in agreement with our observation that short pre-treatment of SBI had a better response in reducing PSI than the similar treatment starting at a later stage. ULK1 has been suggested as a central node that integrates “information” coming from different signaling pathways such as AMPK and rapamycin complex 1 (mTORC1) (40). AMPK positively regulates autophagy induction through ULK1 phosphorylation while mTORC1 act primarily in the opposite way. We here showed a major AMPK-dependent phosphorylation of ULK1, was markedly suppressed in the epidermis from both patients and IMQ-treated mice. In line with these findings, inactivation of AMPK has been found in the lesional epidermis from psoriasis patients (41). Other mechanisms like numerous micro-RNA have also been reported to target ULK1 (21) or the phosphorylation of ULK1 *via* regulating AMPK/mTOCR1 balance (42). Further research is warranted to probe the mechanism whereby ULK1 is mediated in psoriatic KCs.

Taken together, our findings suggest a possible self-regulatory process by downregulating ULK1 to maintain skin homeostasis in psoriasis *via* interfering with keratinocyte-neutrophil interplay. ULK1 inhibitor might be a potential option for treating or preventing relapse of psoriasis.

## Data Availability Statement

The datasets presented in this study can be found in online repositories. The names of the repository/repositories and accession number(s) can be found below: https://www.ncbi.nlm.nih.gov/geo/, GSE166388.

## Ethics Statement

The studies involving human participants were reviewed and approved by research ethics board of Sun Yat-sen Memorial Hospital. The patients/participants provided their written informed consent to participate in this study. The animal study was reviewed and approved by Sun Yat-Sen University Animal Care and Use Committee.

## Author Contributions

ZS, XQ, LZ, and MH performed and analyzed the experiments. ZS and LW drafted the manuscript, designed the experiments, and reviewed the manuscript. XL and DH assisted in the neutrophil-keratinocytes co-culture system and assisted in analyzing the data. GT contributed conceptually to the project and assisted in manuscript preparation. SH assisted in animal experiments of the preventative and therapeutic model and manuscript preparation. CT and ZT gathered and processed serum samples from patients. All authors contributed to the article and approved the submitted version.

## Funding

This study was supported by the National Natural Science Foundation of China [grants 81872524, 82073431] and Guangdong Basic and Applied Basic Research Foundation [grant numbers 2020A1515110320].

## Conflict of Interest

The authors declare that the research was conducted in the absence of any commercial or financial relationships that could be construed as a potential conflict of interest.

## Publisher’s Note

All claims expressed in this article are solely those of the authors and do not necessarily represent those of their affiliated organizations, or those of the publisher, the editors and the reviewers. Any product that may be evaluated in this article, or claim that may be made by its manufacturer, is not guaranteed or endorsed by the publisher.

## Abbreviations

AMPs, anti-microbial peptides; AMPK, AMP-activated protein kinase; ANOVA, analysis of variance; Atg, autophagy‐related gene; CQ, chloroquine; H&E, hematoxylin and eosin; IMQ, imiquimod; KCs, Keratinocytes; PsD, psoriasiform dermatitis; PSI, psoriasis index score; siRNA, small interfering RNA; ULK1, 51-like kinase 13-MA, 3-methyladenine.
