# Supplementary material for: ULK1 Inhibition as a Targeted Therapeutic Strategy for Psoriasis by Regulating Keratinocytes and Their Crosstalk With Neutrophils
Source: Front Immunol. 2021 Aug 4;12:714274. doi: 10.3389/fimmu.2021.714274 (PMC8371267; doi:10.3389/fimmu.2021.714274)
Supplement: Supplementary file 1 [file DataSheet_1.docx]

**Supplementary figures**

**
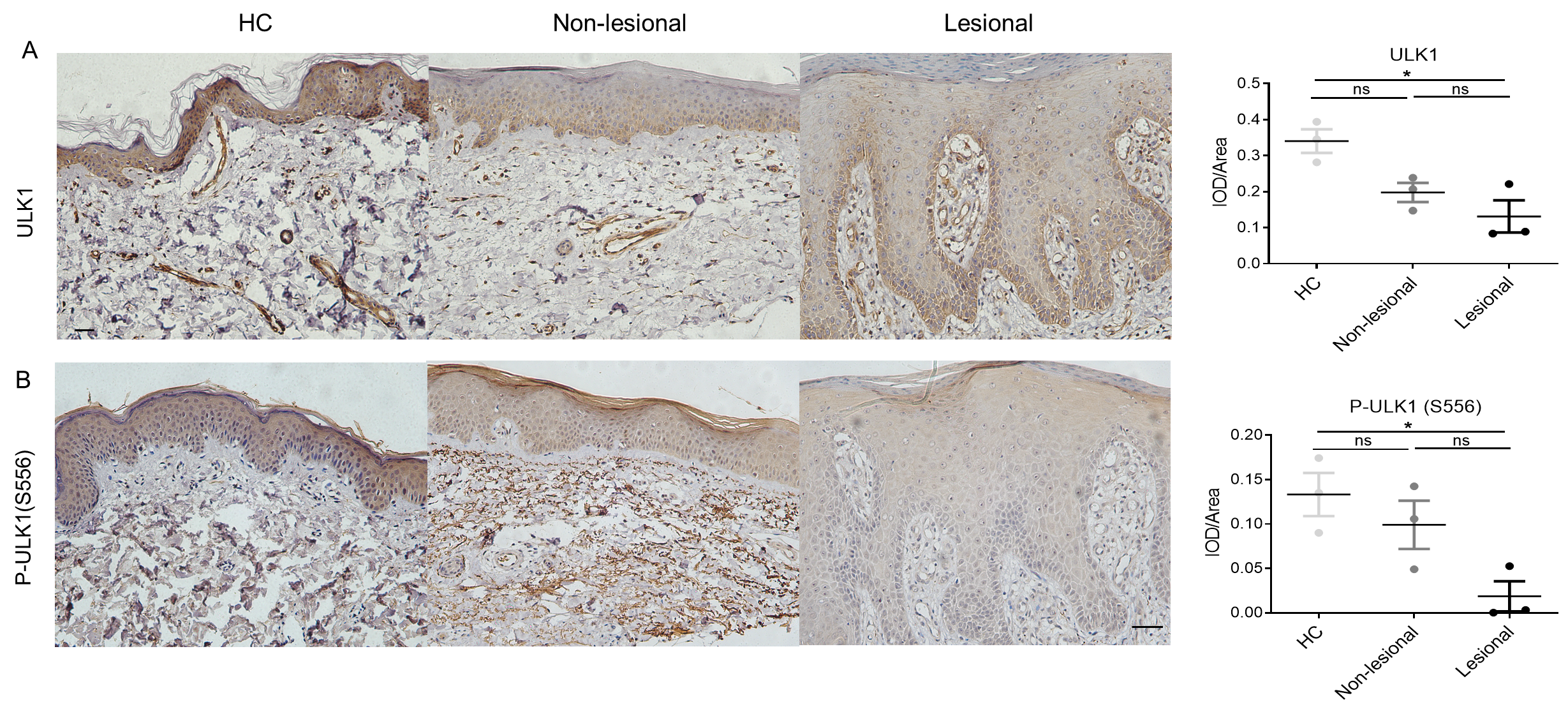
**

**Supplementary figure 1. Expression of ULK1 in non-lesional skin from psoriasis patients.**

Representative image and quantification of ULK1 (A) and phospho-ULK1 (Ser556) (B) expression by immunohistochemical (IHC) staining in healthy control, non-lesional skin and lesional skin from patients psoriasis (n=3).Scale bars, 25 μm. Data are presented as mean± SEM.*p < 0.05.

**
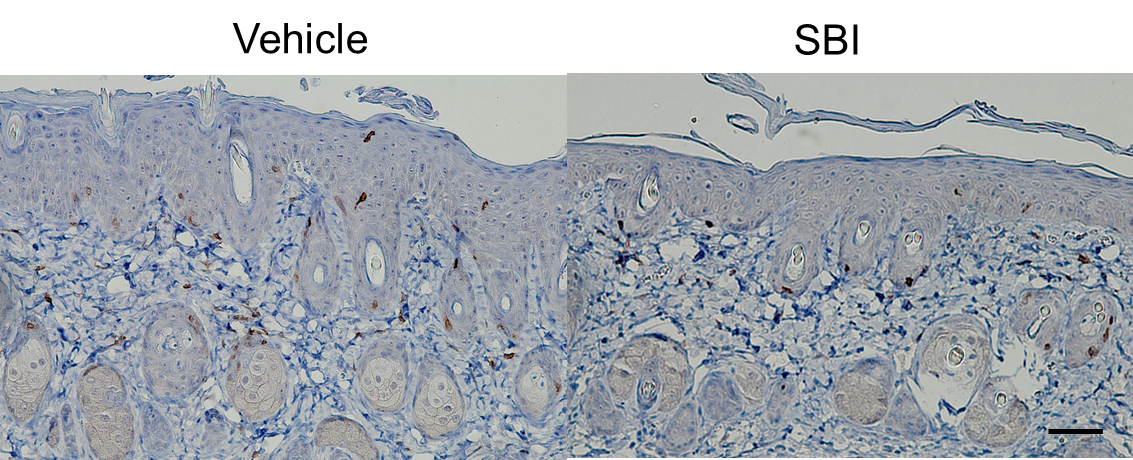
**

**Supplementary figure 2. Comparable infiltration of CD3+ T cell in skin between vehicle or SBI treated mice after IMQ application. Scale bars, 50 μm.**


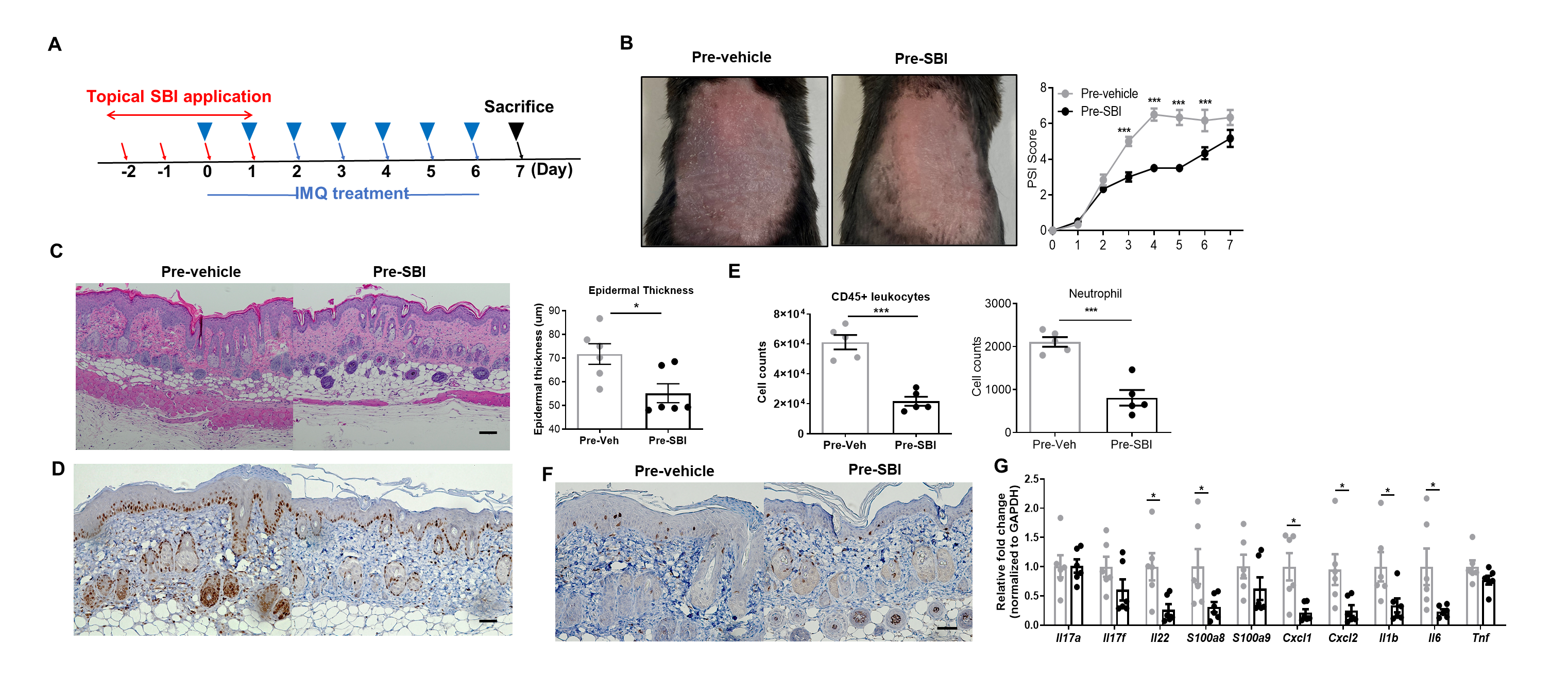


**Supplementary figure 3. Pretreatment with topical SBI delayed IMQ-induced psoriasiform dermatitis**

(A) Schematic illustration of experimental protocols. (B) Manifestations and the severity of skin inflammation measured by PSI score in mice treated as shown in (A). (C) HE staining and histological analysis of epidermis thickness. (D) Ki-67 staining examined by immunohistochemistry staining. (E) Absolute number of infiltrating CD45+ cells and neutrophils per 0.7cm*0.7cm lesional skin.(F) Infiltration of CD3+ T cells examined by immunohistochemistry staining. (G) mRNA expression of cytokines in the whole ear. 6 animals per group. Scale bars, 50 μm. Data are presented as mean± SEM.*p < 0.05; ***p< 0.001.

**
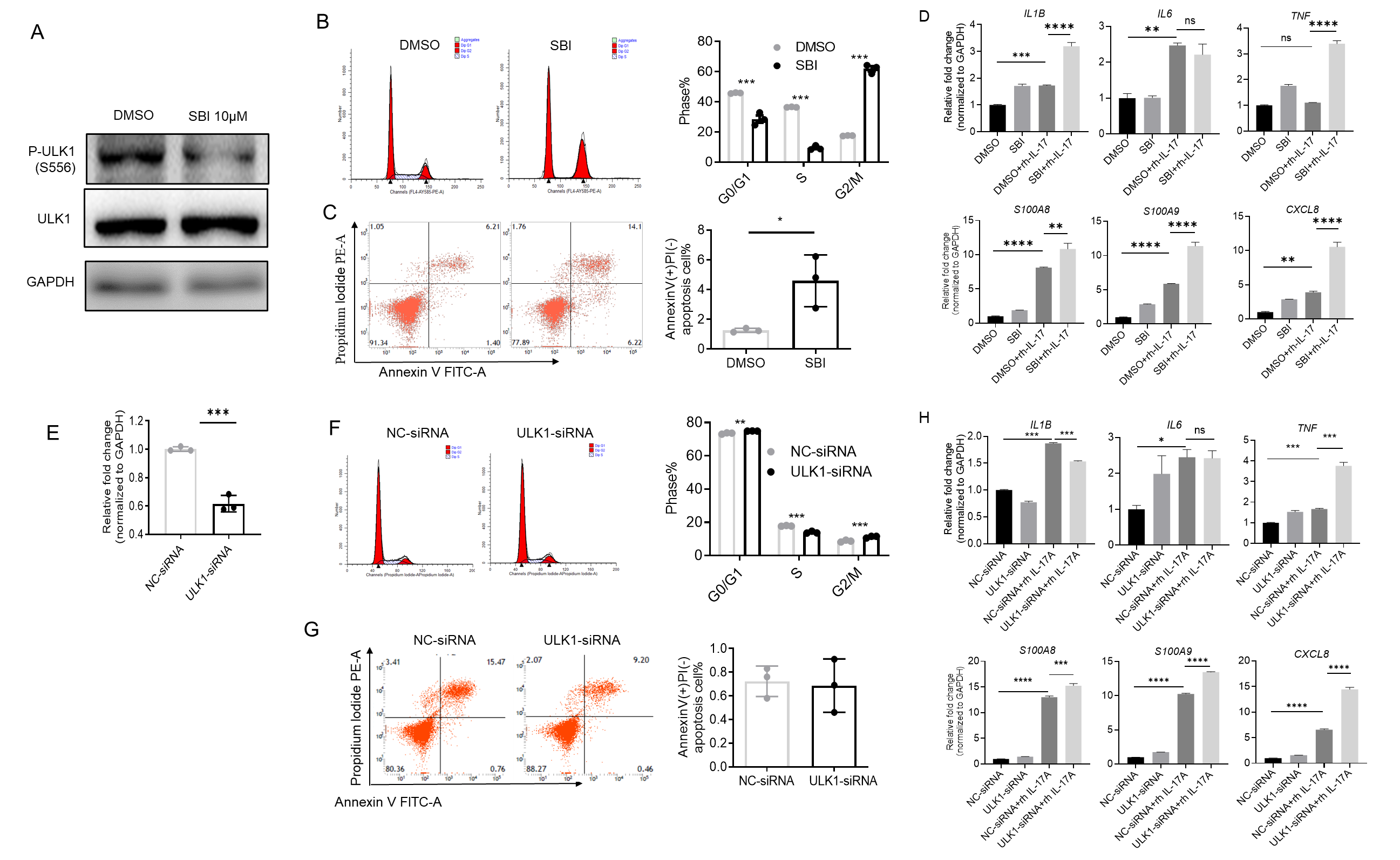
Supplementary figure 4. Proliferation and apoptosis of primary human keratinocytes (PHK) with dysfunction of ULK1**

(A) Expression of p-ULK1(Ser556) and in PHK cocultured with DMSO, SBI-0206965 (SBI) for 24 hours. (B) Cell cycle analysis of PHK 24 hours after cocultured with DMSO or 10 µM SBI-0206965 (SBI). The bar graph shows the percentage of cells in each phase of cell cycle. (C) Apoptosis of keratinocytes 24 hours after serum deprivation in the presence of SBI. (D) mRNA expression of psoriasis-related inflammatory mediators in PHK cocultured with DMSO or 10 µM SBI for 24 hours in the absence or presence of IL-17A (50ng/ml). (E) mRNA expression of ULK1 in PHK transfected with negative control-siRNA(NC-siRNA ) or ULK1-siRNA. (F) Cell cycle analysis of PHK 72 hours after transfection. The bar graph shows the percentage of cells in each phase of cell cycle. (G) Apoptosis of transfected PHK 24 hours after serum deprivation. Data are representative of three independent experiments. (H) mRNA expression of psoriasis-related inflammatory mediators in PHK transfected with NC-siRNA or ULK1-siRNA in the absence or presence of IL-17A (50ng/ml) for 24 hours. Data are presented as mean± SEM.*p < 0.05; **p < 0.01, ***p < 0.001.


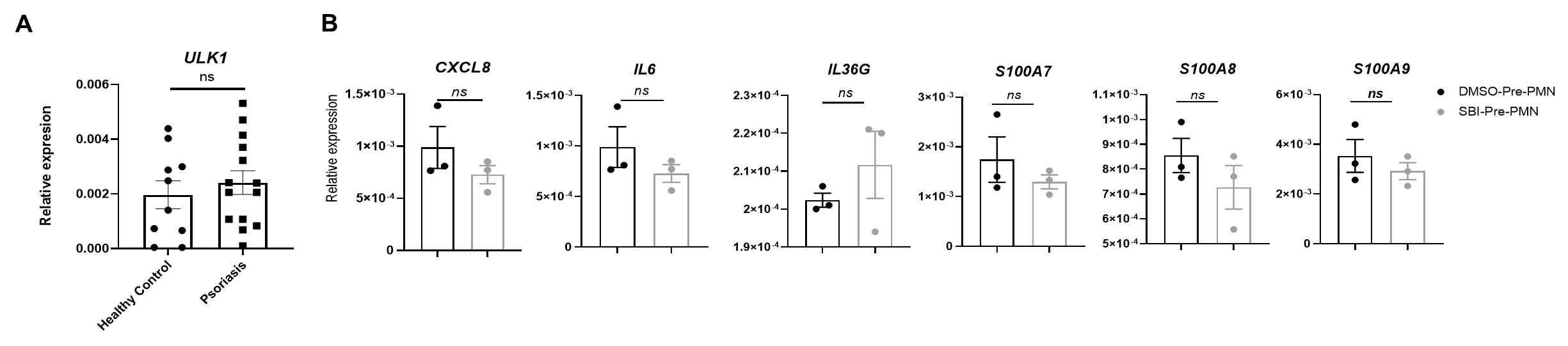


**Supplementary figure 5. Inhibition of ULK1 on neutrophils failed to suppress inflammation in keratinocytes.**

(A) mRNA expression of ULK1 in neutrophils from healthy donors (HC) and psoriasis patients.(B) Neutrophils isolated from HC were pretreated with 10 µM DMSO or SBI for 2hours,and then cultured with HaCat keratinocytes. mRNA expression of psoriasis-related inflammatory mediators in HaCat keratinocytes were examined at 8 hours after co-culture. Data are presented as mean± SEM. Ns, not significant.


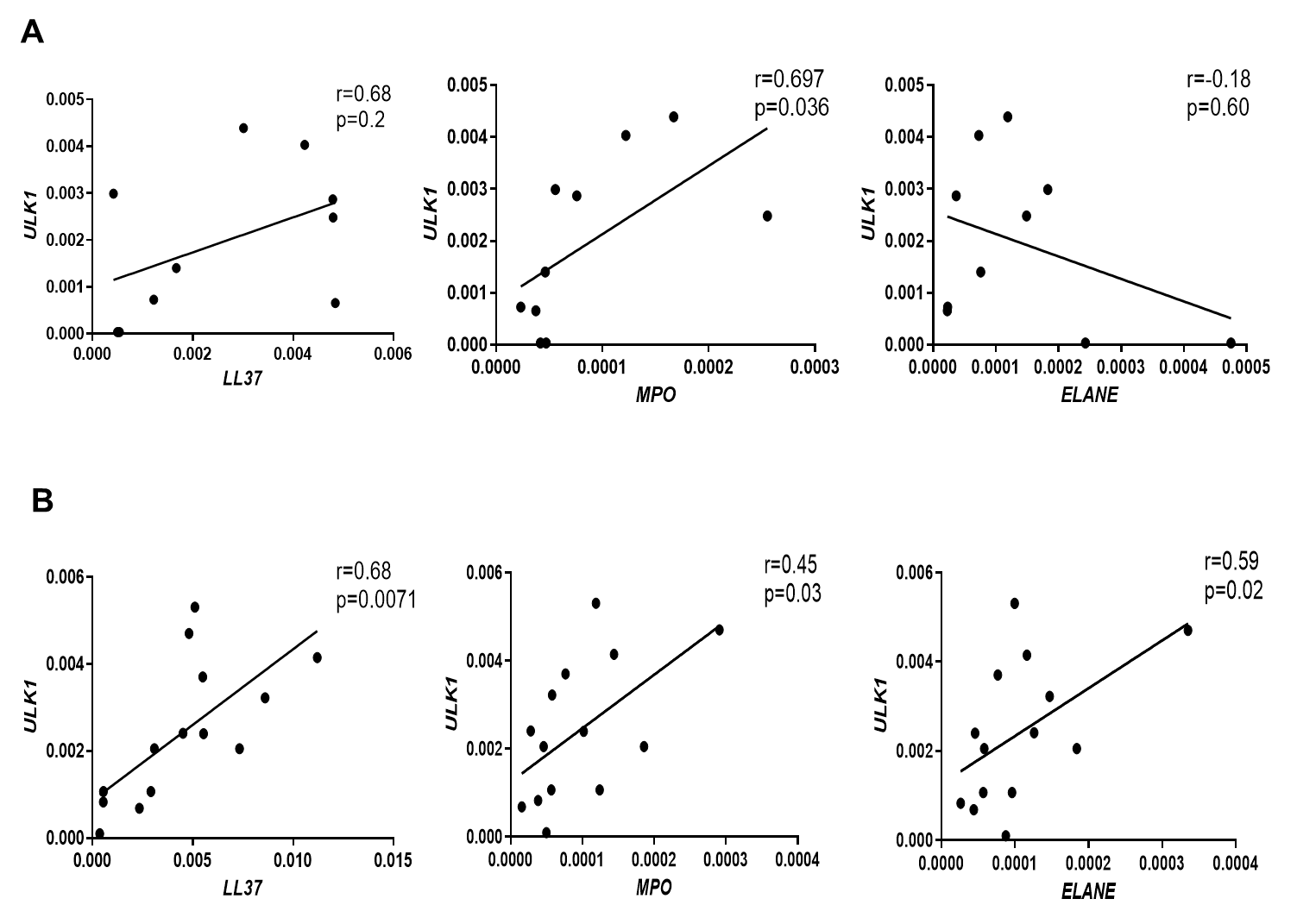


**Supplementary figure 6. Correlation of ULK1 with NETs-related markers in neutrophils from healthy controls (HC) and psoriasis patients**

The mRNA levels of ULK1, LL37, MPO or ELANE were analyzed by q-PCR. Correlation analysis of ULK1 with LL37, MPO or ELANE in neutrophils from HC (A) or psoriasis patients (B). Data are analyzed by spearman rank correlation test.


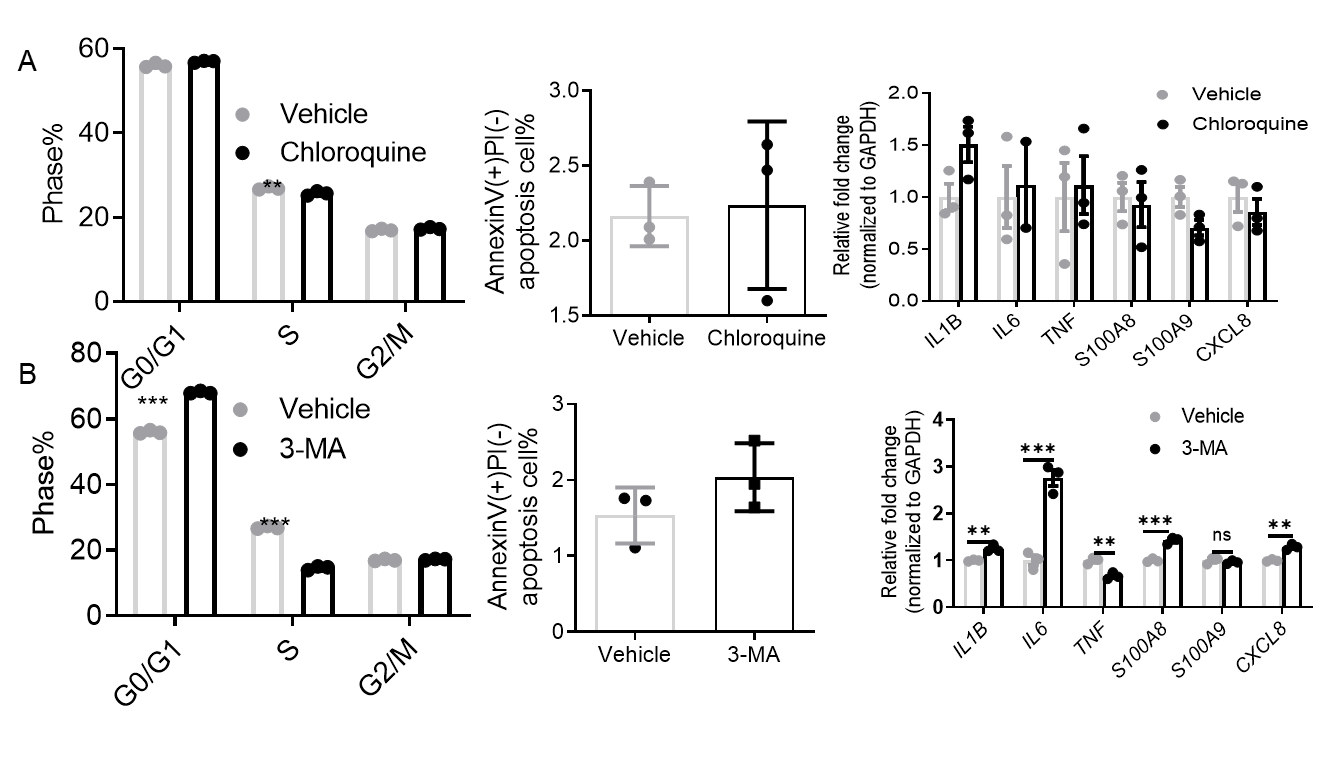
**Supplementary figure 7. Autophagy inhibition impaired the proliferation without affecting apoptosis or suppressing inflammation of primary human keratinocytes (PHK)**

Cell cycle, apoptosis analysis and mRNA expression of psoriasis-related inflammatory mediators by keratinocytes at 24 hours after treatment with 10µM chloroquine (A) or 5mM 3-MA (B). Data are representative of three independent experiments. Data are presented as mean± SEM.*p < 0.05; **p < 0.01; ***p< 0.001. ns=not significant.

**Supplementary figure 8. Effect of 3-MA on regulating inflammatory marker of primary human keratinocytes (PHK) in the presence of IL-17A**

Expression of psoriasis-related inflammatory mediators by PHK at 24 hours after with coculture with IL-17A (50ng/ml) in the presence of vehicle or 5mM 3-MA. Data are representative of three independent experiments. Data are presented as mean± SEM.*p < 0.05; **p < 0.01; ***p< 0.001.

**Supplementary methods**

**Biopsy site of skin**

| Biopsy site | HC | Ecezema | psoriasis |
| --- | --- | --- | --- |
| Neck/Scalp | 0 | 3 | 2 |
| Trunk/HiP | 2 | 4 | 1 |
| thigh | 0 | 0 | 2 |
| lower leg | 5 | 2 | 5 |
| Upper limps | 1 | 1 | 0 |

**Cell cycle analysis and apoptosis analysis of keratinocytes**

For cell cycle analysis, cells were harvested 24 to 72 hours after siRNA transfection, 10µM SBI-0206965 (MedChemexpress, Princeton, NJ, USA), 10µM chloroquine (Sigma-Aldrich, St. Louis, MO, USA) and 5mM 3-MA treatment, respectively (Sigma-Aldrich, St. Louis, MO, USA), fixed by adding drop-sized cold 70% ethanol, and then pelleted and resuspended in propidium iodide solution (Sigma-Aldrich, St. Louis, MO,USA) and analyzed by flow cytometry.

For Annexin V assay of cell apoptosis, transfected keratinocytes and keratinocytes treated with the indicated compounds were cultured in serum-free medium for 24 hours. Cells were then harvested by trypsin, washed twice with PBS and stained according to the manufacturer's instructions for the Annexin V-FITC Apoptosis assay kit (Thermo Fisher Scientific, Waltham, MA, USA). Both apoptotic cells and live cells were detected by flow cytometry (BD Biosciences, San Jose, CA, USA).

**Sample preparation for flow cytometry**

Anti–mouse CD45 (30-F11), CD11b (M1/70) and Ly6G (IA8) antibodies were purchased from BioLegend (San Diego, CA). Whole ear skin was minced and digested with Liberase TM (Roche, Mannheim) and DNase I (Sigma-Aldrich) with addition of 5% fetal bovine serum to obtain whole skin cell suspensions before passing tissue through a 70-um cell strainer. Single-cell suspensions from the cervical draining or popliteal lymph nodes were prepared by mashing the tissue through a 70-um cell strainer.. Anti-mouse CD16/32 (BD Biosciences, San Jose, CA) was added to cells prior to staining to block binding to Fc-receptors.

**PCR primer**

The primers were pre-designed (otherwise the sequence are shown) and obtained from Integrated DNA Technologies, Inc (Skokie, IL, USA) or synthesized according to the sequence from Primerbank database. rimers:

| Gene name | Identifer or sequence |
| --- | --- |
| Gapdh | Mm.PT.39a.1 |
| *Il17a* | Mm.PT.58.6531092), |
| *Il17f* | Mm.PT.58.9739903), |
| *Il22* | F:5’-ATG AGT TTT TCC CTT ATG GGG AC-3’,  R: 5’-GCT GGA AGT TGG ACA CCT CAA-3’ |
| *Cxcl1* | Mm.PT.58.42076891 |
| *Cxcl2* | Mm.PT.58.10456839 |
| *S100a8* | Mm.PT.58.44003402 |
| *S100a9* | Mm.PT.58.41787562 |
| *Il1b* | Mm.PT.58.41616450 |
| *Il6* | Mm.PT.58.10005566 |
| *Tnf* | Mm.PT.58.12575861 |
| *S100A8* | F:5’-ATG CCG TCT ACA GGG ATG AC-3’,  R: 5’-ACT GAG GAC ACT CGG TCT CTA-3’ |
| *S100A9* | F:5’-GGT CAT AGA ACA CAT CAT GGA GG-3’,  R: 5’-GGC CTG GCT TAT GGT GGT G-3’ |
| *CXCL8* | F:5’- TTT TGC CAA GGA GTG CTA AAG A-3’,  R: 5’-AAC CCT CTG CAC CCA GTT TTC-3’ |
| *IL1B* | F:5’-ATG ATG GCT TAT TAC AGT GGC AA-3’,  R: 5’-GTC GGA GAT TCG TAG CTG GA-3’ |
| *IL6* | F:5’-ACT CAC CTC TTC AGA ACG AAT TG-3’,  R: 5’-CCA TCT TTG GAA GGT TCA GGT TG-3’ |
| *TNF* | F:5’-GAG GCC AAG CCC TGG TAT G-3’,  R: 5’-CGG GCC GAT TGA TCT CAG C -3’ |
| *ULK1* | F:5’-GGC AAG TTC GAG TTC TCC CG-3’,  R: 5’-CGA CCT CCA AAT CGT GCT TCT-3’ |
| *CXCL1* | F:5’-AAC CGA AGT CAT AGC CAC AC-3’,  R: 5’-GTT GGA TTT GTC ACT GTT CAG C-3’ |
| *CXCL2* | F:5’-GCT TGT CTC AAC CCC GCA TC-3’,  R: 5’-TGG ATT TGC CAT TTT TCA GCA TCT T-3’ |
| *CXCL5* | F:5’- AGC TGC GTT GCG TTT GTT TAC-3’,  R: 5’- TGG CGA ACA CTT GCA GAT TAC-3’ |
| *IL36G* | F:5’- AGG AAG GGC CGT CTA TCA ATC-3’,  R: 5’- CAC TGT CAC TTC GTG GAA CTG-3’ |
